# Serious illness care Programme UK: assessing the ‘face validity’, applicability and relevance of the serious illness conversation guide for use within the UK health care setting

**DOI:** 10.1186/s12913-019-4209-8

**Published:** 2019-06-13

**Authors:** Tamsin McGlinchey, Stephen Mason, Alison Coackley, Anita Roberts, Maria Maguire, Justin Sanders, Francine Maloney, Susan Block, John Ellershaw, Peter Kirkbride

**Affiliations:** 1 Palliative Care Institute Liverpool, University of Liverpool, Ground floor Cancer Research Centre, 200 London Road, Liverpool, L3 9TA UK; 2 Clatterbridge Cancer Centre NHS Foundation Trust, Wirral, UK; 3 Ariadne Labs, Brigham and Women’s Hospital and Harvard T.H. Chan School of Public Health, Boston, MA USA

**Keywords:** Serious illness, Communication, Intervention, Person Centred, Care planning

## Abstract

**Background:**

When doctors have honest conversations with patients about their illness and involve them in decisions about their care, patients express greater satisfaction with care and lowered anxiety and depression. The Serious Illness Care Programme (the Programme), originally developed in the United States (U.S), promotes meaningful, realistic and focused conversations about patient’s wishes, fears and worries for the future with their illness. The Serious Illness Conversation Guide (the guide) provides a framework to structure these conversations. The aim of this paper is to present findings from a study to examine the ‘face validity’, acceptability and relevance of the Guide for use within the United Kingdom (UK) health care setting.

**Methods:**

A multi-stage approach was undertaken, using three separate techniques:

1. Nominal Group Technique with clinician ‘expert groups’ to review the Serious Illness Conversation Guide: 14 ‘experts’ in Oncology, Palliative Care and Communication Skills;
2. Cognitive Interviews with 6 patient and public representatives, using the ‘think aloud technique’; to explore the cognitive processes involved in answering the questions in the guide, including appropriateness of language, question wording and format
3. Final stakeholder review and consensus.

**Results:**

Nominal Group Technique

Unanimous agreement the conversation guide could provide a useful support to clinicians. Amendments are required but should be informed directly from the cognitive interviews. Training highlighted as key to underpin the use of the guide.

Cognitive interviews

The ‘holistic’ attention to the person as a whole was valued rather than a narrow focus on their disease. Some concern was raised regarding the ‘formality’ of some wording however and suggestions for amendments were made.

Final stakeholder review

Stakeholders agreed amendments to 5/13 prompts and unanimously agreed the UK guide should be implemented as a part of the pilot implementation of the Serious Illness Care Programme UK.

**Conclusion:**

Use of the guide has the potential to benefit patients, facilitating a ‘person-centred’ approach to these important conversations, and providing a framework to promote shared decision making and care planning. Further research is ongoing, to understand the impact of these conversations on patients, families and clinicians and on concordance of care delivery with expressed patient wishes.

## Background

The need for improved clinician communication and a more ‘patient centred’ approach to care delivery is consistently highlighted in government policy within the United Kingdom (UK) [1–4] and research literature [5–7]. Clinicians frequently fail to involve seriously ill patients in discussions and decisions regarding their current and future care, resulting in limited opportunity to engage in conversations about hopes, fears and concerns with their clinical team [1, 2, 8]. Poor clinician-led communication can engender or exacerbate psychological distress for patients with serious illness and their families [9–11]; indeed ‘staff attitudes’, ‘behaviour‘and ‘poor communication’ account for the majority of complaints about the National Health Service (NHS) [12, 13]. A recent report from the Royal College of Physicians (RCP) ‘Talking About Dying’ highlighted significant barriers to initiating conversations about prognosis and treatment choices, including: clinician confidence and personal fears about having these conversations; a culture of targets and organisational pressures within the NHS; and a belief that a culture exists among the public to avoid discussing death [4].

The Serious Illness Care Programme (“the programme”) is a multi-component, systems-based intervention designed to improve the care of all persons with serious illness [14]. Serious illness is defined as: “a health condition that carries a high risk of mortality and negatively impacts a person’s daily function or quality of life, excessively burdens their caregivers, or both” [15]. Developed by Ariadne Labs in the United States (U.S.) the Programme promotes meaningful, realistic and focussed conversations about the patients fears, worries and wishes for the future with their illness and consists of three elements: 1) clinical tools, including a specially developed Serious Illness Conversation Guide (‘the guide’) using ‘patient tested language’, to ensure these important issues are addressed consistently and sensitively; 2) expert training and coaching for members of the multidisciplinary team in how to have these conversations; 3) systems innovations and Electronic Medical Records (EMR) modifications, whereby organisations create clear processes to identify patients and document details derived from the conversation in order to share it with other health professionals. The shared information then informs ongoing care and treatment planning and promotes ongoing conversations [14].

Preliminary data from a large cluster randomised controlled trial among cancer patients at the Dana Farber Cancer Institute in Boston, showed implementation of the Serious Illness Care Programme leads to “more, earlier and better conversations” by oncologists; lowered anxiety and depression in patients; and high levels of patient satisfaction and positive behaviour change resulting from the conversation [16, 17]. A recently published study in primary care patients also suggests that the Serious Illness Care Programme promotes more and better conversations, as well as the need for further research [18].

In response to continued governmental challenges to improve communication and care for patients with serious illness [19, 20], NHS England provided funding in 2016/17 for a service improvement project to pilot implementation of the Programme at three clinical sites; two primary care, one cancer centre. In preparation for the pilot, a three stage research project was undertaken to explore the ‘face validity’, applicability and relevance of the clinical tool, the Serious Illness Conversation Guide, to explore whether adaptations were required for the UK before its use in the pilot. This paper describes the process and outcomes of this study.

## Methods

A multi-stage approach was taken in order to engage a wide range of stakeholders; healthcare professionals and members of a patient and public involvement (PPI) group from within one cancer centre in the North West. The research was structured into three stages:

### Stage 1 - Nominal Group Technique (NGT) [21, 22]

A variation of a small-group discussion to reach consensus. The Nominal Group Technique (NGT) provides a structured format to collect and synthesise multiple views from different groups or individuals and commonly involves 4 stages: silent generation of ideas, round robin, clarification of ideas and voting. Three ‘expert groups’ made up the participant groups and the following structure, based on the 4 stages, was used to guide the discussion and achieve consensus:

1. Generation of ideas: each individual group reviewed the guide and considered the following discussion points:

- *Discussion Point 1:* What do you think about the overall utility of incorporating the Serious Illness Care Guide into clinical practice? Consider:

- Timing of discussions;
- Incorporating this guide into conversations during clinical consultations;
- Planning for future care.

- *Discussion Point 2:* For each ‘prompt’, consider the following:

- What do you think of how the questions are worded?
- Are there any phrases/concepts within any of these questions that may be problematic?
- Is there anything currently missing from this list of prompts, that you feel would be important to discuss/consider at this time?

2. Round robin: each group feedback the ‘ideas’ generated from the individual group discussion.
3. Clarification: Points raised by individual groups (concerns/problems identified or suggestions for amendment) were then opened up for discussion in the wider group.
4. Voting: Consensus was gained for each discussion point and suggestion for amendment. Consensus opinion was recorded on flip charts, written up following the meeting, and circulated to all members for accuracy and confirmation of content.

Sample: participants for the NGT were recruited from three healthcare professional groups with expertise in Oncology, Palliative Care and Communication Skills. Clinicians were sent an information sheet to explain the purpose of the meeting and invite them to take part. Consent to participate was gained following clinician ‘sign up’ and subsequent attendance at the meeting.

### Stage 2 - cognitive interviewing

In depth one-to-one cognitive interviews were undertaken with lay representatives, as a proxy for the patient or ‘user’ perspective, to explore the cognitive processes involved in answering the questions in the guide, including appropriateness of language, question wording and format [23, 24].

Sample: Participants were recruited from patient and public representative groups from within the cancer centre, with experience of living with or caring for a loved one with cancer. Specifically, the researcher attended a Patient Council meeting within the cancer centre, where the purpose of the research was introduced. During the meeting, permission was given for the researcher to send a participant information sheet (PIS) to members of the Patient Council and other lay representative groups located in the cancer centre. Following this, and giving at least 24 h for consideration of the information, the researcher made contact with individual members to ask if they wished to take part. If they agreed to take part, then a time and place convenient to the participant was arranged.

Due to the in-depth nature of the interviews that were undertaken, the ‘richness’ of the data generated (information power), and the specific focus of the ‘phenomena’ (the guide), it was felt that the sample of 6 lay representatives would meet the objectives of the study [25].

Five interviews were undertaken within the participants own home, and one interview was undertaken in a quiet room within the cancer centre identified by the lay participant. The Cognitive Interview itself was guided by the ‘think aloud’ process [26], which encouraged participants to articulate their thoughts (or ‘think aloud’) as they read through the guide. The interviews did not use a specific topic guide; participants were given the Serious Illness Conversation Guide (the guide) and asked to read each prompt aloud and talk through their understanding, including how they might formulate an answer. This process also enabled words or concepts that participants were unsure of to be highlighted and discussed. Each interview was audio recorded and then transcribed verbatim to aid analysis. Thematic Analysis was undertaken, in order to generate key themes from the data, using the “substance of the interview” to interpret “meanings and perceptions created and shared during a conversation” [27].

Written, informed consent to participate was gained from all participants, via a consent form. Ethical approval for this element was received from Wales Research Ethics Committee 5 (Bangor), reference number 16/WA/0062.

### Stage 3 – final stakeholder review and consensus of the serious illness care guide

Multiple stakeholders reviewed results from stage 1 and 2 before achieving final agreement on suggested amendments to the guide. This meeting was integrated into the scheduled Steering Group Meeting held as part of the service improvement project, funded by NHS England, to adapt the Serious Illness Care Programme for use in the UK. Consent to participate was gained through attendance at the meeting. All stakeholders were sent written information on the purpose of the meeting, which was to gain consensus on the final amendments to the Serious Illness Conversation Guide. Stakeholders at this meeting included members from: Patient Council (lay members); NHS England (policy makers); Ariadne Labs (research and health professional members); Marie Curie Palliative Care Institute Liverpool (research and health professional members) and Clatterbridge Cancer Centre (research and health professional members). All members in attendance at the meeting participated in this element.

## Results

**Fig. 1:**
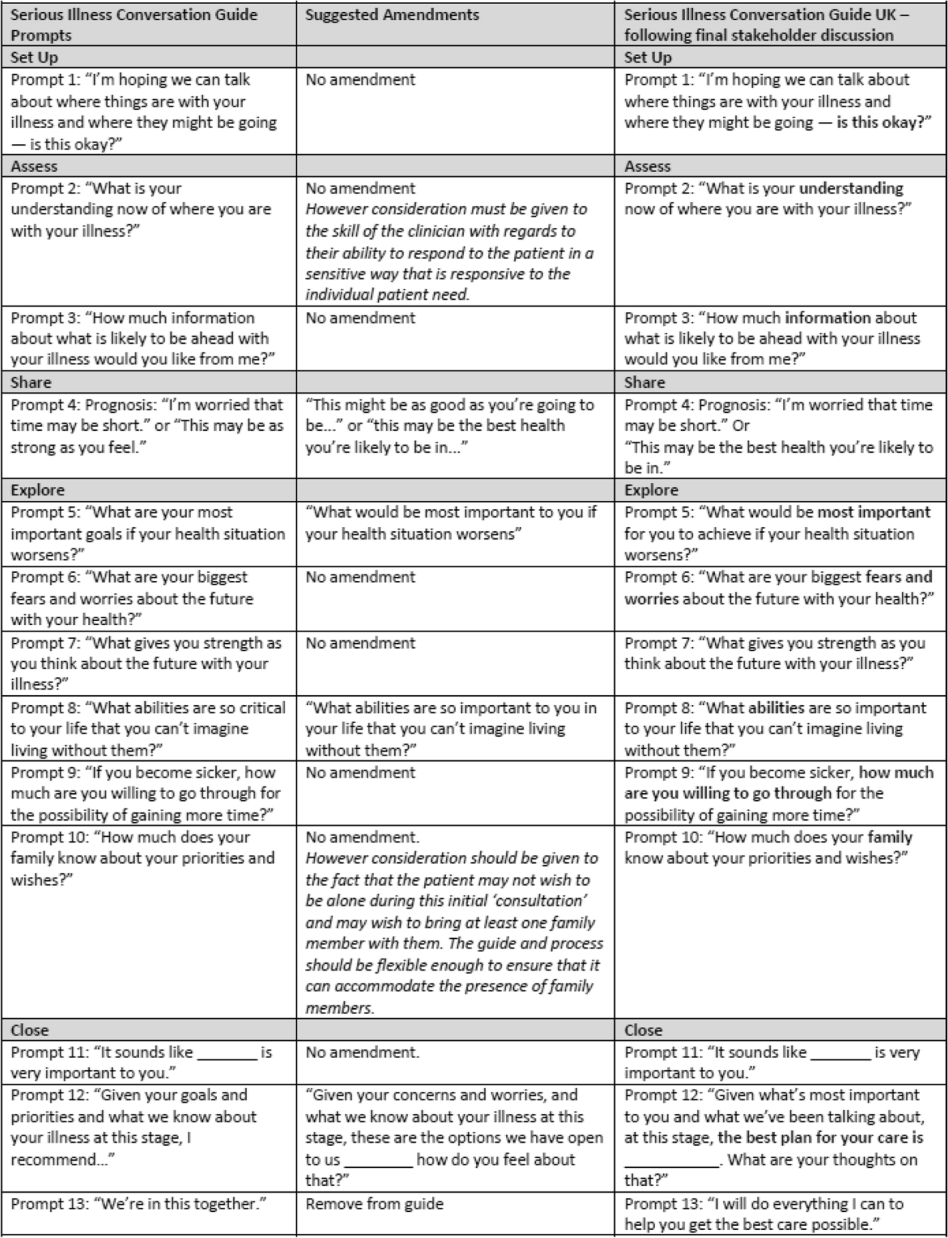
Serious Illness Conversation Guide – Overview of Final Amendments

The main findings from all three stages are presented in this section. Figure 1 below illustrates the process of amendment following the results of the project, and includes the UK adapted prompts, following the final stakeholder discussion meeting.

### Stage 1 - Nominal Group Technique (NGT)

14 participants attended:

- 5 Consultant Medical Oncologists
- 4 palliative care experts: 3 Consultants in Palliative Medicine and 2 Specialist Registrars in Palliative Medicine
- 4 communication skills experts: 3 Senior Lecturers in communication skills (all RGN) and 1 Consultant in Palliative Medicine with a lead role in communication skills training for undergraduate medical education.

Results: Agreed discussion points from group discussion:

1. Acceptability, applicability and relevance of the Serious Illness Conversation Programme:

- *Serious Illness Conversation Guide:* Oncologists valued this, viewing it as a structure to initiate, engage and support in serious illness conversations. They indicated that they would derive confidence in initiating these conversations, knowing the language in the guide was ‘patient tested’. Views from the communication skills and palliative care ‘expert’ groups were that the guide was potentially reductionist, due to the ‘scripted’ format, however they acknowledged this may be due to their increased clinical experience in handling these types of conversations.
- *Education and training:* The importance of a robust programme of education and training, incorporated into the implementation of the programme, was highlighted. All groups emphasised the need for ongoing training and support throughout the implementation period, for example, by employing coaching and mentoring to help clinicians overcome initial anxieties and address challenges in the change to current practice.
- *Systems innovations:* All ‘expert’ groups agreed that adherence to the systems innovations (infrastructure and monitoring) as part of the overall Programme was essential to underpin use of the guide and support clinicians to have conversations with their patients.

2. Wording of the Serious Illness Conversation Guide: discussion/suggestion for amendment

- All members of the Nominal Group agreed it would be appropriate to leave suggestions for wording changes until after analysis of the results from the cognitive interviews with PPI representatives. All members felt that the perspectives of this group, as a proxy for the patient ‘user perspective’, would ensure that any suggestions for amendment would be rooted in the ‘patient voice’.

The group unanimously agreed that, pending modifications informed by cognitive interviews, the guide would effectively support clinicians in initiating high quality serious illness conversations.

The final consensus from the NGT was that amendments to the guide should not be put forward by this group. Instead, suggestions for wording amendments should be underpinned by the ‘rich’ data that would be generated by the Cognitive Interviews (stage 2) with lay representatives.

### Stage 2 - cognitive interviews

Six lay representatives took part, including two male and four female participants. 5 overarching themes emerged from the analysis:

1. *Promoting a partnership approach to care planning conversations*
2. *Importance of flexibility of approach and ability to incorporate the prompts as part of a ‘natural’ conversation*
3. *‘Opening the door’ – Importance of ongoing conversations throughout the patient journey*
4. *Education and training essential as part of the implementation of the programme*
5. *‘Formality’ of some words/phrases a concern: goals, priorities and wishes, abilities, critical*

#### 1. Promoting a partnership approach to care planning conversations

Most participants described the guide as a useful ‘conversation piece’, empowering the patient to begin to think about, and talk through, what is most important to them in regards to their future care:

*“...it starts a conversation...it’s an aide memoir…you can use the guide and...you’ll go off script undoubtedly, because conversations will take a different turn, and you can’t anticipate, but it allows you to make sure you...start here and you finish here, and by the time you’ve finished, you have covered everything...you know...” (P1, male)*

Responses also suggested that this should not be a one off conversation. For the majority of participants, this promoted a ‘partnership approach’ to conversations and decision making, so that information and care planning can be tailored to individual patient need:

*“I mean, “how much information of what is likely to be ahead” ... everything, because as far as I’m concerned it’s a partnership, you’re not abdicating responsibility to somebody to do whatever they want to you, you want to know what they’re going to do. You’re part of the decision making process...” (P2, female)*

Participants also described the value they saw in the attention to exploring the patients ‘holistic’ needs as an individual, rather than exclusively focusing on their ‘disease’. This was important to all participants:

“*It’s seeing the person as an individual and that they’ve got a life.” (P2, female)*

#### 2. Importance of flexibility of approach and ability to incorporate the prompts as part of a ‘natural’ conversation

There was concern the guide could create an artificial ‘interview’ situation, suggesting that clinicians should be able to ‘adapt’ the language and format to be responsive to what is important to the patient. The ‘formality’ of some of the language in the guide was a concern for some, particularly the impact this could have on the ability to have a ‘natural’ conversation:

*“...I think sometimes using these words just makes it sound like a formal interview...you need...to be able to put the patient into a situation where they feel comfortable to be able to open up with their concerns...and saying to them, what are your “goals”, or what are your “fears and worries”, or what “strength” just makes it sound as though you’re sitting across from the desk, from somebody in an office, having an interview” (P1, male)*

One participant in particular highlighted the importance of ensuring information given by the patient is respected, taken on board and actively incorporated into the ongoing care of that patient. Failing to engage could undermine the patient centred aim:

*“...they need to acknowledge [what’s important]…when [the patients]…say it as well... [regarding a care plan] one of the things for me, one of the key things was about using some natural stuff and so I told them, they knew, but then every time it was mentioned it was like eyes rolling”. (P2, female)*

The quote above reinforces the perspective that the guide should not be seen as a ‘script’ from which deviation is not possible. Clinicians should be able to adapt or amend their conversation as necessary, and it is important that the patient’s wishes are respected:

*“...what if patients start talking about it in a different order...I’m not going to sit there and watch somebody tick a box, talking to me about I’m dying...” (P5, female)*

#### 3. ‘Opening the door’ – importance of ongoing conversations throughout the patient journey

Implementation of the UK Programme should reinforce to clinicians that this is not a one off conversation, and that follow up is essential:

*“If you’re going to have this sort of conversation, that’s only the beginning...you’ve then got to be prepared to follow up what the patient says, haven’t you, and be able to...it’s a starter isn’t it...” (P2, female)*

The same participant emphasised that some patients may not wish these conversations to happen in isolation and that as well as ongoing dialogue, involvement of the patients family and friends should be facilitated if that is the patients wish:

*“They need to know before this point, how that person wants that information, or, if they want that information... erm...or if they want a friend to have, you know, their loved one to have that information or what that...but that needs to be done” (P2, female)*

#### 4. Education and training essential as part of the implementation of the programme

Education and training were viewed as key in the implementation and continued use of the guide in practice. Participants in both the patient interviews and the nominal group recommended that education and training must emphasise to clinicians the integration of guide prompts into a ‘natural’ conversation. This view is underlined by the quote below, from a patient with concerns that use of the guide and engaging in the conversation may not come ‘naturally’ to some clinicians:

*“...but the most important thing is the clinician, because there are some who can never do that in a million years because it’s not in their nature...it [use of the guide] could make it [conversation] considerably worse...it would be a very uncomfortable experience” (P2, female)*

The quote above illustrates the individual skill of the clinician is vital when it comes to using the guide, and that use without adequate training could be detrimental to patient care. This indicates a role for general communication skills training in addition to the guide to ensure that clinicians have the necessary skills to ensure patients are able to engage fully with the conversation, and ask their own questions in return:

*“...and they [patient] need to be able to have the facility to be able to ask questions and one of the difficulties I found with some clinicians, not all of them...is that they can be erm... difficult...less than communicative when it comes to patients wanting to question them... and that’s the training down to the doctors.” (P1, male)*

#### 5. ‘Formality’ of words or phrases is a concern

For the majority of participants, the word ‘goals’ was identified as a problem; it was too formal and they didn’t initially understand the intention behind asking this prompt. Participants felt that the formality of the word ‘goals’ is often associated with an organisational context, rather than within a sensitive conversation with a patient, and could therefore be prohibitive to a ‘natural’ conversation:

*“Goals...would they see it as a goal? I don’t know whether they’d see it as a goal...it’s more about love isn’t it, love for the family...it doesn’t have a feeling of wanting to see my first grandchild, that’s not a goal.” (P5, female)*

*“I don’t like the certain phrases which makes it sound far too stilted and formal...so you know, if you can soften the language...so that it makes them, makes somebody feel more comfortable, then...I would have no problem at all” (P3, female)*

While the wording and meaning of most questions were understandable and acceptable to the participants, one was almost uniformly singled out for removal by members of both groups: “we’re in this together:”

*“...I think the last sentence is a bit naff...cos you’re not actually in it together...if somebody said that to me I’d go “pardon”...you’re not, I mean yes put some comforting words there but...we’re in it together is hardly comforting, when you know it’s not true.” (P5, female)*

### Stage 3 – final stakeholder review and consensus of the serious illness conversation guide UK

Results from stages 1 and 2 were presented to the group, alongside suggested wording amendments to the guide which were identified from the Cognitive Interviews. All thirteen individual ‘prompts’ on the guide were reviewed, using facilitated discussion. Figure 1 illustrates the main discussion points and confirmation of the final amendments to the guide, following consensus agreement. Five prompts were subsequently amended. All stakeholders at the meeting agreed this would be the final version of the UK guide. Stakeholders also unanimously agreed that the UK guide could now be implemented as a part of the pilot implementation of the Serious Illness Care Programme UK.

## Discussion

Results from this study suggest that the adapted Serious Illness Conversation Guide has the potential to support clinicians to have conversations with seriously ill patients regarding their current and future care, and use of the guide has the potential to promote shared decision making and identification of what matters most to patients. Further, the guide provides a good foundation from which to improve the way important conversations about future care planning are undertaken, documented and used to improve care planning and decision making. This study also highlighted useful information about the way the guide might be used in practice, as well as how it is implemented as part of the Serious Illness Care Programme UK.

Through the cognitive interviews, participants identified that the guide could benefit patients by facilitating a more ‘person-centred’ approach to these important conversations, although some cited their own negative experiences of clinician communication as a reason for their positive reaction to the guide and the programme. Good communication and patient satisfaction with care is often dependent on the existing relationship between patient and clinician; with trust, honesty and empathy being the qualities most appreciated by patients [28]. Studies have shown that communication skills training which employs the use of communication ‘tools’ can improve clinicians ‘responsiveness’ to patient’s concerns and engender more ‘empathic’ encounters with patients following training [9, 29]. While participants uniformly valued the use of the guide, the structured approach, although also seen as a benefit, was identified as a potential weakness, particularly if used/adopted by clinicians that had not undergone training and followed the guide by rote. Findings from stage 1 and 2 both highlight the guide should not act as a ‘script’ that cannot be deviated from or adapted as necessary, according to individual patient needs and wishes. For example, during the cognitive interviews, participants commented that the clinician should be able to ‘adapt’ the language as part of a natural conversation, rather than reading verbatim as if in an ‘interview’ situation. Education and training must, therefore, be a key part of the implementation of the UK programme, and in supporting the sustainable use of the guide within any organisation.

It has been argued that engaging in honest discussion about difficult information may empower patients with regards to decision-making as well as enhance rather than diminish hope [30, 31]. This sentiment was reflected in findings from this study, where participants described the guide as a useful ‘conversation piece’, which would allow the patient to think about, and talk through, what is most important to them in regards to their current and future care. This was an important element for participants and they also identified that the guide, with its focus on exploring the patients’ ‘holistic’ needs, treats patients as individuals with ‘a life’ rather than exclusively focussing on their ‘disease’. Delaying important discussions about the future when prognosis is poor can subsequently preclude the identification and achievement of a patient’s preferences for end-of-life care [32], which also highlights the value that a tool such as the guide could bring to the improvement of patient care.

Although the majority of prompts were viewed positively, discussions from stage 1 and 2 highlighted concerns with some of the language within the guide. For example, concern was felt regarding the more ‘formal’ language within the guide, in particular the use of the word ‘goals’. The consensus was that this word may not translate particularly well, as the word ‘goal’ is often associated with an organisational context rather than within a sensitive conversation with a patient. Given the overall feedback received as part of this project, prompt 5 was then amended to remove ‘goal’. While other comments from stage 1 and 2 identified other minor concerns with ‘formal’ words and phrases such as ‘what gives you strength’ and ‘fears and worries’, these terms were not identified as problematic and were therefore not amended. However, given the concerns participants had, it is imperative that clinicians are able to integrate the prompts into a ‘natural’ conversation. It is recommended that general training in communication skills for clinicians, as well as in use of the tool, is essential for the implementation and ongoing use of the programme. Without this, the potential for challenges and variation of quality in the delivery of the serious illness conversation may occur.

The final amendment was to the concluding prompt ‘we’re in this together’; unanimous agreement from ‘expert’ groups and lay representatives, this prompt would not translate to the UK context and should be removed from the guide. This prompt received the strongest negative reaction, particularly from the lay representatives in this study. Concerns were raised that this could appear ‘superficial’ and ‘disingenuous’ on the part of the clinician, potentially undermining the ‘person centred’ approach to this conversation and the relationship that the clinician may have built up with the patient. Following the final stakeholder review, it was felt that the conversation guide did need an additional prompt to reaffirm a commitment to the ongoing care and support of the patient, and alternative wording was agreed: ‘I am here to make sure you get the best care possible’.

## Conclusion

Use of the guide to support conversations between clinicians and patients with serious illness has the potential to improve ‘person-centred’ care, and facilitate improved care planning through shared decision making, to ensure greater concordance between documented wishes and patient outcomes. Although the guide, and the programme as a whole, was viewed as a positive intervention, further robust research and evaluation on its effect in clinical practice was recommended to develop the evidence base prior to any local or national ‘roll out’. It was suggested that research should also include in-depth ‘user experience’ of the guide in practice. Specifically qualitative and quantitative methods should be used to unpack patient and family perception and understanding of engaging in serious illness conversations, as well as clinician perception of using the guide to support their conversations. Following this study pilot implementation of the Programme was undertaken in 3 NHS sites, two primary care sites and a cancer centre. In line with the findings from this study, further research is ongoing. A feasibility study is currently being undertaken to explore use of the Programme within the cancer centre, and results from this study will inform any further potential roll out of the Programme.

## Data Availability

The data of this study are available from the corresponding authors on reasonable request.

## Abbreviations

NGT: Nominal Group Technique
NHS: National Health Service
the guide: The Serious Illness Conversation Guide
the Programme: The Serious Illness Care Programme
U.S.: United States
UK: United Kingdom
